# A Review and Survey of Local Eastern Kentucky Medicinal Plants and Their Pharmacological Benefits

**DOI:** 10.3390/plants14203182

**Published:** 2025-10-16

**Authors:** Pratyusha Veldhi, Chris Crager, Ayesha Ghayur, Zaheer Ul-Haq, Muhammad Nabeel Ghayur

**Affiliations:** 1 Kentucky College of Osteopathic Medicine, University of Pikeville, Pikeville, KY 41501, USA; pratyushaveldhi@upike.edu (P.V.); chriscrager@upike.edu (C.C.); ayeshaghayur@upike.edu (A.G.); 2 Dr. Panjwani Center for Molecular Medicine and Drug Research, International Center for Chemical and Biological Sciences, University of Karachi, Karachi 75270, Sind, Pakistan; zaheer.qasmi@iccs.edu

**Keywords:** ethnopharmacology, herbs, North American folk medicine, Pike County, plant extract, traditional use

## Abstract

Medicinal plants are used all over the world to prevent, cure, and manage many different diseases. The aim of this study was to provide knowledge on different medicinal plants that are native to Pike County, Eastern Kentucky, USA. The study involved two stages of activity. First, it involved a survey of some county locals to identify medicinal plants used for different medical purposes. The second part dealt with searching research databases like PubMed and Google Scholar to find out if any of those plants, identified in the survey, have any published scientific studies on them. The results of the survey identified 14 locally used medicinal plants (*Asimina triloba*, *Callicarpa americana*, *Chimaphila umbellate*, *Cichorium intybus*, *Eupatorium perfoliatum*, *Monotropa uniflora*, *Paulownia tomentosa*, *Phytolacca americana*, *Portulaca oleracea*, *Sassafras albidum*, *Ampelopsis glandulosa*, *Ulmus rubra*, *Verbascum thapsus*, and *Xanthorhiza simplicissima*) belonging to different families, plant types and used for a wide variety of purposes. Most plants belonged to the Ericaceae and Asteraceae families, were mostly herb type, while the most common plant part was berries, leaves and roots. The survey also showed that the local population use these plants for a variety of purposes, such as a food additive, insect repellant, antirheumatic, antiarthritic, coffee alternative, laxative, antitussive, analgesic, or anti-infective. Sometimes these plants and plant substances are used raw, made into tea, or even made into an edible jam product. For the second part of the study, all the plants were supported by multiple published studies. The most common pharmacological activity among the plants was antimicrobial, followed by anticancer, antioxidant and anti-inflammatory activities. Eastern Kentucky is well known for its scenic Appalachian Mountains, but the area holds potential for innovative herbal medicine as well. More interest and research are needed to further explore the treasure of medicinal plant use knowledge resting in this area. Additionally, more phytopharmacological and phytochemical studies are needed to investigate the scientific potential of traditionally used medicinal herbs from this region.

## 1. Introduction

The use of medicinal plants has been a cornerstone of traditional medicine systems worldwide, providing natural remedies for various ailments throughout human history [1,2]. The World Health Organization previously initiated a comprehensive effort to catalog all known medicinal plants globally, resulting in the identification of over 20,000 species [3]. Specifically, Southern folk medicine in the USA employs various treatment approaches, incorporating herbs, foods, prayers, and restorative actions to promote healing [4]. Rooted in a holistic mind–body–spirit approach, it embraces dualistic principles and emphasizes balance and interconnectedness [5].

**Figure 1:**
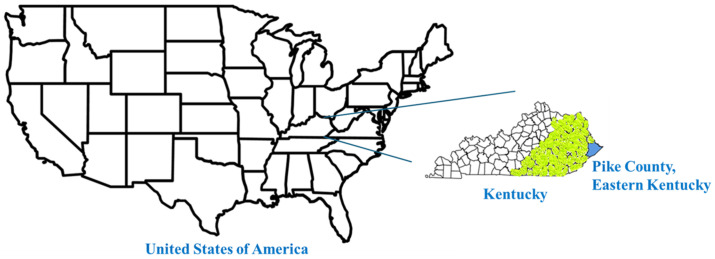
Map showing Pike County in Eastern Kentucky (in blue), the United States of America (USA), and all of the Kentucky counties that constitute Central Appalachia (Pike County + all counties in yellow). The study involved the survey and review of the pharmacological benefits of medicinal plants from Pike County, Kentucky, USA.

In the Central Appalachian region of Eastern Kentucky (Figure 1), there exists a rich heritage of botanical medicine that has been passed down through generations of both indigenous peoples and settler communities. Appalachian forest plants have been integral to drug manufacturing since colonial times. For generations, gathering these wild medicinal resources has provided residents of the region with an essential source of additional income [6]. The unique geographical and climatic conditions of this region have given rise to a diverse array of plant species with significant therapeutic potential [7]. Recent studies have shown that plants from this region contain bioactive compounds with antimicrobial, anti-inflammatory, and antioxidant properties [8].

The growing interest in natural products and the increasing resistance to conventional antibiotics has led to renewed scientific attention on traditional medicinal plants [9,10]. While medicinal plants may seem like mere “old wives’ tales” or simple home remedies, their significance was demonstrated during the recent COVID-19 pandemic [11]. This highlights that, despite their ancient origins, medicinal and herbal practices remain relevant in modern times. Eastern Kentucky’s flora represents an untapped reservoir of potential therapeutic agents, with many species containing novel compounds that could address current healthcare challenges. Modern analytical techniques have enabled researchers to identify and characterize these bioactive compounds, providing scientific validation for traditional uses while discovering new applications [12,13]. Rather than looking at analytical techniques, in this study the authors plan to survey the use of medicinal plants and then perform a literature review of those plants obtained as part of the survey. As a result, this study aims to systematically document and analyze 14 medicinal plants native to Pike County in Eastern Kentucky (coordinates 37.4793° N, 82.5188° W), examining their traditional uses, pharmacological properties, and potential therapeutic applications.

## 2. Study Design

A survey was undertaken in Pike County, Eastern KY (Figure 1). The consent forms and the research methodology for the survey were reviewed and approved by the Institutional Review Board of the University of Pikeville (IRB Protocol # 25_0022). The objectives of the study, along with any related risks for participating in the survey were clearly stated and conveyed to the participants.

An initial attempt was made to locate and identify local healers, herbalists or elders with knowledge of local medicinal plant use and knowledge of folk medicine in Central Appalachia (Figure 1). After many attempts and efforts, asking around and calling places, only a total of four local individuals were identified who had some degree of local traditional medicinal plant use knowledge. All of these four individuals were employees of the University of Pikeville. All of these individuals are local to this area and their lineage in Central Appalachia goes back for generations. The source of their knowledge was the use of medicinal plants in their families by their elders, which is why they know which medicinal plant is used in what form and for what indication. All the interviewed individuals were asked these following basic questions to obtain the data:

1. Can you remember or know of a local Eastern Kentucky plant used in your household for a medicinal purpose?
2. Do you know the name of that plant (local/common name)?
3. Do you know what part of that plant (fruit, leaf, root, etc.) is used for its benefit?
4. Do you know the clinical or medical indication for which it is used?
5. Do you know how the plant is prepared, including any special instructions, for using it for its medicinal purpose?

The interviewees provided information like local/common names of plants, medicinal use, plant part used and the mode of preparation of the medicinal plant. For the other plant information, like scientific names, families that those plants belonged to, and plant type, some of these local printed resources were used [14,15,16,17].

The second part of the study involved determining whether some of those local medicinal plants (identified in a survey of local Eastern Kentuckians) have been studied scientifically for their pharmacological benefits. For this purpose, we searched databases like PubMed and Google Scholar to find evidence of published studies. Each plant was searched by using its scientific name (Table 1), any alternate scientific name(s) (Table 2), and local/common names (Table 1). The inclusion criteria focused on studies that discussed the pharmacological activities, therapeutic benefits, or mechanisms of action of these plants or their bioactive compounds. Experimental (in vitro, in vivo), clinical studies, and review articles were included only if they contained substantial discussion of pharmacological evidence related to traditional medicinal uses. There were no restrictions on the year of publication; articles from any period were considered eligible for review. The literature search combined general pharmacological and ethnomedicinal terms with the scientific, alternate, and common names of the plants identified in the local survey. The main keywords used included “pharmacology,” “traditional use,” “medicinal use,” “mechanism,” and “benefits,” in combination with the specific plant names (for example, “Plant name AND pharmacology” or “Plant name AND traditional use”). Boolean operators (AND, OR) were applied where appropriate to refine and expand the search results. Search results were screened by title and abstract to identify studies that met the inclusion criteria. Full texts of potentially relevant articles were then reviewed to confirm eligibility and inclusion into the study.

Studies were excluded if they focused primarily on botanical, agricultural, or ecological aspects of the plants, such as taxonomy, cultivation, or environmental distribution, without addressing their pharmacological or therapeutic application. Non-English publications and reports without available abstracts or full texts were also excluded from the review.

## 3. Results and Discussion

**Figure 2:**
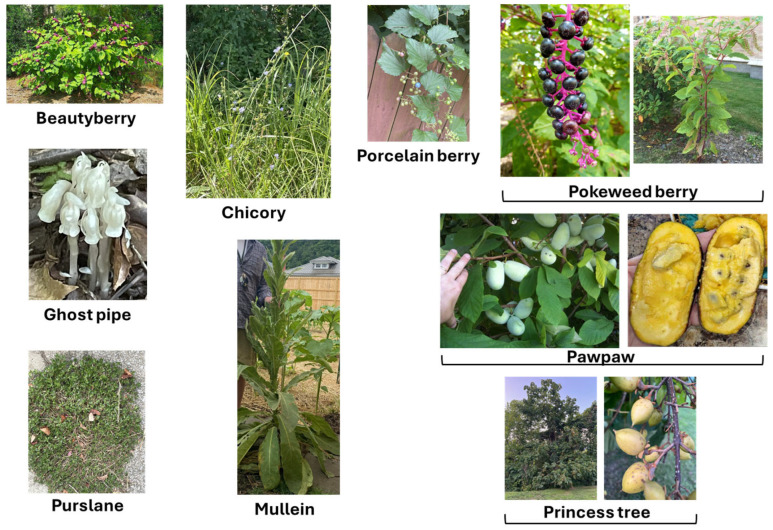
Pictures of some of the plants reviewed and discussed in this project. All plants were pictured in or around Pike County, Eastern Kentucky (pictures taken by author C.C.).

**Figure 3:**
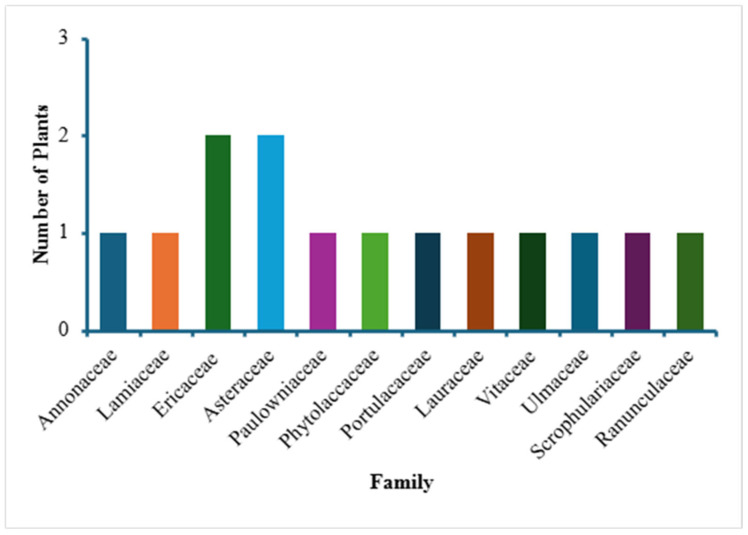
A bar graph exhibiting the various plant families represented by the medicinal plants discussed in this review.

**Figure 4:**
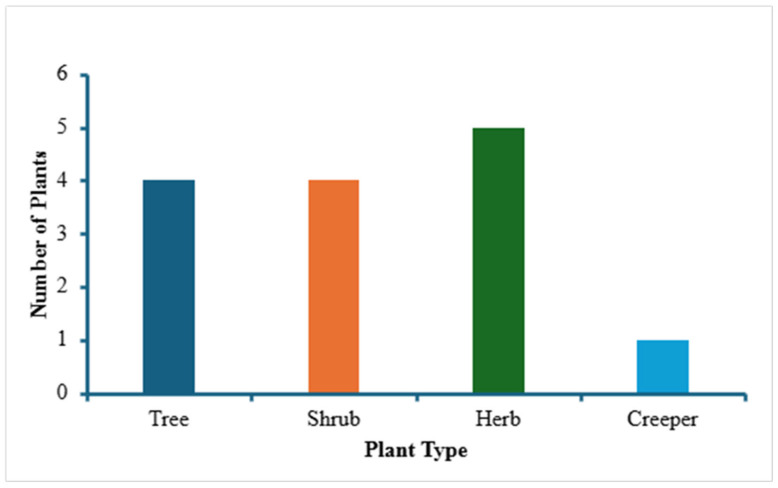
Bar graph showing the distribution of the 14 medicinal plants under study, based on their plant type.

**Figure 5:**
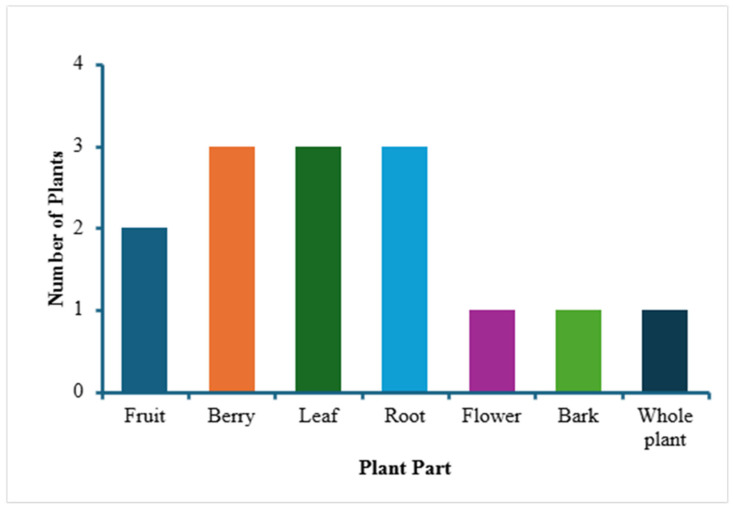
Bar graph demonstrating the various medicinal plant parts used by locals in Eastern Kentucky for their medical benefits.

The first part of the study (the survey and the answers obtained as a result of the survey of locals in Pike County) led to the identification of 14 different locally used medicinal plants, listed in Table 1. Pictures of some of these 14 plants are given in Figure 2, all were taken in and around Pike County and identified by one of the authors (C. C.). Table 1 shows information about the scientific name and family of the plants, their plant type (creeper, herb, shrub, tree), local/common names, medicinal uses/indications, the plant parts used and the mode of their preparation. When the data from this Table 1 were further analyzed, it was noted that, of all of the medicinal plants identified in the survey, most belonged to two families—Ericaceae and Asteraceae (Figure 3). Based on plant type, most of the plants are herbs followed by trees and shrubs (Figure 4). In terms of the part of the plant used for obtaining the medical benefits, the most common part is berries, leaves and roots, followed by fruits, flowers, bark and whole plant (Figure 5). The survey showed that the local population uses these plants for a variety of medicinal purposes, such as a food additive (pawpaw, princess tree), insect repellant (beauty berry), antirheumatic (pipsissewa), antiarthritic (pokeweed, porcelain), coffee alternative (chicory), laxative (boneset, slippery elm), antitussive/expectorant (boneset, mullein), analgesic (ghost pipe), and anti-infective (yellowroot). Most of these plants, identified as part of the survey, are considered safe for human consumption. It can be seen from Table 1 that they are used for medicinal purposes and consumed, as these are generally considered safe. Either these plants are eaten raw (pawpaw fruit, princess tree fruit, purslane leaves) or processed, as with pokeweed, whose seed can be harmful unless rendered consumable by boiling; berries, which are made into a jam; yellowroot, which is made into a liquid; and many others (chicory root, boneset leaves, slippery elm bark and mullein), which are prepared into a tea.

**Figure 6:**
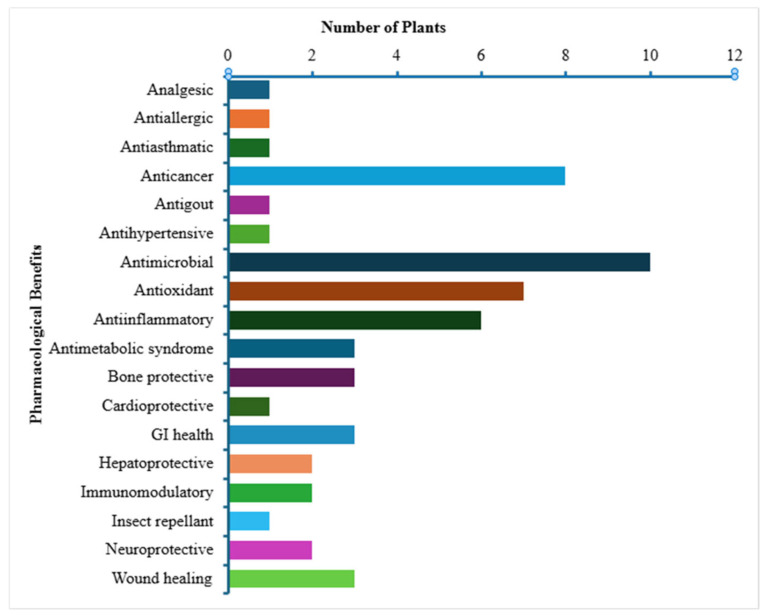
Graph showing the various pharmacological benefits of the 14 Eastern Kentucky medicinal plants under study in this project. Note: Antimicrobials include antibacterial, antiviral, and antiparasitic activities; gastrointestinal (GI) health includes GI tone modulation, effect on digestion, and overall GI benefits; antimetabolic syndrome includes antidiabetic effect.

Once the plants were identified, as mentioned above, they were searched in credible resources like PubMed and Google Scholar to see if there are any scientific reports published related to them, using the scientific names, alternate scientific names and common names (Table 1 and Table 2). This part of the research showed that all of the plants have a variable number of published papers relating to them, and in consideration of a wide variety of pharmacological activities. Table 3 lists these different activities, while Figure 6 shows a comparison of the most common pharmacological activities among all of these plants. The analysis revealed that antimicrobial activity was the most frequently reported activity followed by anticancer, antioxidant and anti-inflammatory effects (Figure 6).

For detailed insight into general and specific folk use (Central Appalachia and Eastern Kentucky) and the published pharmacology and phytochemistry of these plants, each plant is discussed below in detail.

### 3.1. Asimina triloba (L.) Dunal

*A. triloba*, colloquially known as pawpaw, is a small tree, found in eastern North America, primarily throughout Kentucky and Ohio [15,16,17]. Pawpaw can be used for anti-aging, tumor suppression, antioxidant, and antimicrobial purposes [18]. Our survey of locals from Pike County in Eastern Kentucky revealed that the fruit of the plant is considered nutritious and is eaten by locals (Table 1).

The unripe fruit of pawpaw contains phenolic compounds and exhibits antioxidant activity. This source of antioxidants has the potential to delay the aging process [31]. Hypoxia-inducible factor-1 (HIF-1) is a transcription factor that activates target genes promoting tumor cell survival and adaptation. It also induces tumor angiogenesis through VEGF, a target for many anticancer drugs such as sorafenib. Pawpaw crude extract inhibits HIF-1, which correlates with the suppression of VEGF and GLUT1, demonstrating its potential as an anticancer alternative medicine [32]. A 95% ethanol extract of ripe pawpaw has shown sensitivity against *Corynebacterium xerosis* and *Clostridium perfringens*, indicating its potential as an antimicrobial [19]. Other groups have also shown an anticancer potential of fruit extract of pawpaw [33,34,35].

### 3.2. Callicarpa americana L.

*C. americana* is commonly known as beautyberry or American beautyberry. This small shrub is native to North America, specifically the southern USA (cultivated in Kentucky) and northern Mexico [16,19]. A decoction of the roots and branches of beautyberry is used by the Alabama tribe of Native Americans in rheumatism and fever [20]. Our own survey of Eastern Kentucky locals led to the information that berries from the plant are used for their insect repellant activity (Table 1).

*C. americana* has shown promise, as per the published data, in restoring beta-lactam sensitivity to treat MRSA infections, treating acne vulgaris, and repelling insects such as ticks, mosquitoes, and arthropods [36,37,38]. MRSA is typically treated with vancomycin, which is known for its adverse effects, such as nephrotoxicity and red man syndrome [152]. A chloroform-soluble extract from the combined fruits, leaves, and twigs of *C. americana* demonstrated synergy with fluoroquinolones against MRSA by downregulating efflux pumps associated with multidrug resistance [36]. Acne vulgaris is a common skin condition that affects young adults and contributes to low self-esteem and an approval-seeking nature [153]. Current treatments for acne include benzoyl peroxide, salicylic acid, antibiotics, retinoids, and oral contraceptives [154]. However, the daily use of antibiotics, such as erythromycin and clindamycin, has led to drug-resistant strains of *Cutibacterium acnes* [155]. *C. americana* leaf extracts exhibited MICs against *C. acnes* isolates at very low concentrations [39], proving their efficacy in reducing bacterial proliferation and inflammation. *C. americana* produces bioactive diterpenoids that have insect-repellent activities [37,38,40]. These diterpenoids are derived from kolavenyl diphosphate (KPP), which is formed by Class II diterpene synthases (diTPS). Spathulenol, intermedeol, and callicarpenal are the terpenoids responsible for repelling insects. This proves the use of this plant, by the locals, as an insect repellant.

### 3.3. Chimaphila umbellata (L.) Barton

This shrub belongs to the Ericaceae family, and it is commonly known as pipsissewa. *C. umbellata* is well known for its anti-fungal, antioxidant, and anticancer properties [21]. It was used to treat inflammation of the synovial membrane and ulceration of cartilages as early as 1844 [22]. The findings from our endeavor while interviewing locals here in Pike County, Eastern Kentucky, revealed that leaves are used traditionally for their antirheumatic effects (Table 1).

Chimaphilin is one of the major active components in *C. umbellata* and decreases fungus cell wall, transcription, and mitochondrial functions [21]. Chimaphilin also induces apoptosis in MCF-7 cancer cells and human osteosarcoma cells, providing new avenues for treatment in drug resistant tumor cells [21]. The antioxidant activity of *C. umbellata* crude extract was identified using DPPH (2,2-diphenyl-1-picrylhydrazyl) assay, explaining its role in healing skin wounds [41]. Additionally, the plant has also been shown to inhibit receptor activator of nuclear factor-κB ligand (RANKL), indicating its bone protective effects [42], and possess antimicrobial activity [22,43].

### 3.4. Cichorium intybus L.

This plant, commonly known as chicory (root), is a medicinal plant and a coffee substitute [24]. It is distributed across Kentucky and is abundantly available locally, although it is naturalized from Europe [16]. Infusion of the chicory root is used as a tonic in the native American tradition [20]. Our survey found that tea made from roots of the plant is used as a coffee alternative (Table 1).

The pharmacological properties of *C. Intybus* are hepatoprotective, antiallergic, antitumor, antioxidant, anti-inflammatory, antidiabetic, antimicrobial, and analgesic [44]. One study specifically showed how aqueous, and methanolic chicory seed extract lowered mortality, alkaline phosphatase (AP), aspartate aminotransferase (AST), and alanine aminotransferase (ALT) levels in mice with liver damage [45]. Chicoric acid reduces basal hyperglycemia and corrects insulin resistance, indicating a possibility for its therapeutic application for diabetes [44]. Along with all of the health benefits mentioned above, *C. intybus* also has antiprotozoal activity [46] and the ability to be used as treatment for gout [47,48,49]. Purified *C. intybus* root extracts showed concentration-dependent antiparasitic effects on *Trypanosoma cruzi* [46]. Further studies are needed to understand which metabolites and what mechanisms are responsible for the antiparasitic effect, but *C. Intybus* would likely become a novel anti trypanosomatid agent. Lastly, the plant extract and chicoric acid inhibit the nuclear factor kappa-light-chain-enhancer of activated B cells (NF-κB) and NOD-like receptor family pyrin domain containing 3 (NLRP3) signaling pathways, arresting the release of interleukin (IL)-1β, which is a proinflammatory cytokine that causes symptoms of gout [48]. Employing chicory extract as an anti-gout agent offers a natural, cost-effective alternative to traditional gout medications, potentially reducing adverse effects.

### 3.5. Eupatorium perfoliatum L.

*E. perfoliatum*, commonly known as boneset, grows in wet meadows and along streams across Kentucky [16]. Historically, it has been employed for its antipyretic, anti-inflammatory, and immune-boosting properties to treat conditions such as fever, influenza, and bone pain, often associated with febrile illnesses [23]. Additionally, other traditional uses have also been highlighted, such as diaphoretic, anti-catarrhal [24], digestive, viscera strengthening, restoration of body tone, alterative, antiseptic, cathartic, emetic, febrifuge, diuretic, and astringent [14]. Whole plant infusion of the plant is used in Native American traditions as a tonic for colds, sore throat, and influenza [19,20]. Our own survey revealed that a tea of boneset leaves is credited by locals in Eastern Kentucky with laxative and cough suppressant properties (Table 1).

Pharmacologically, it has been shown to stimulate the immune system and reduce inflammation, making it particularly useful for managing symptoms of viral infections [50], such as those seen in COVID-19 [51,52], and dengue fever [53,54,55]. In addition, there is evidence of the benefits of this plant as a cardioprotective [56], anti-inflammatory [57], and cytotoxic and antibacterial agent [58]. Overall, *E. perfoliatum*’s diverse pharmacological actions make it a valuable herbal remedy in managing fever, inflammation, and viral illnesses.

### 3.6. Monotropa uniflora L.

*M. uniflora*, commonly called ghost pipe due to the shape of its flower, is a non-green herb found in dry to mesic woods across Kentucky [16,17]. Our survey found that the flower of the plant is used traditionally for pain relief (Table 1).

Not many pharmacological studies have been performed on this plant. From one reference, it was reported that a fungus, *Penicillium daleae* L3SO, was isolated from the plant. From this fungus two cage-like polyketides, penidaleodiolide A and penidaleodiolide B were isolated and identified. These compounds showed significant neurotransmission regulation activity when tested on murine neurons [59]. A recent digital survey done on the use of ghost pipe in the USA found that there is widespread use of this plant. The main use is for pain relief, but other uses include treatments related to sleep, relaxation, depression and grief, anxiety, eye inflammation, and spiritual well-being [60].

### 3.7. Paulownia tomentosa (Thunb.) Steud.

*P. tomentosa*, a tree whose fruit is typically sliced and eaten, has been recognized for its pharmacological potential due to its rich phytochemical profile. Commonly known as the princess tree, it is naturalized from China but is now found across all of Kentucky [15,16,17]. Our conversation with the local population revealed that the fruit of this plant is regarded to be nutritious and is in salads (Table 1).

The plant contains bioactive compounds such as flavonoids, phenolic acids, and other secondary metabolites that exhibit various medicinal properties such as antioxidant, antibacterial, and cytotoxic [61]. Studies have highlighted its anti-inflammatory potential, particularly the fruit, which contains compounds that can inhibit inflammatory responses by targeting cytokines IL8, IL6, and human neutrophil elastase activity [62]. Compounds isolated from the fruit of *P. tomentosa* have the potential to be used to develop a new drug to treat airway inflammation. In particular, C-geranylated flavonoids isolated from the fruit have been identified as potent anti-inflammatory agents, showing promise in modulating inflammation at a molecular level [63]. The flavonoids not only reduce the secretion of tumor necrosis factor-α (TNF-α) but also the associated mRNA. A number of other studies have corroborated these findings and have reported the anti-inflammatory activity of the princess tree [64,65,66]. Additionally, the plant is also reported to undertake anticancer [67,68,69,70], antimicrobial [71,72,73,74,75,76], antiviral and antioxidant [77,78], and neuroprotective [79,80] activities.

### 3.8. Phytolacca americana L.

*P. americana*, commonly known as American pokeweed, is found in open woods, fields, and disturbed sites across Kentucky [16,17]. Plant shoots are edible, while the plant can be poisonous. It is medicinally known to be emetic, laxative and with some habit-forming properties [14]. Roots are known to be employed for hemorrhoids while the fruit is useful in rheumatism and sores [14]. Native North Americans report using this plant as a poultice for ulcers while the root infusion is employed in eczema [19,20]. A survey performed with local Eastern Kentucky inhabitants revealed that the berry of this herb is boiled or made into a jam and then used for its antiarthritic effect (Table 1).

Pokeweed has been extensively studied for its wide range of medicinal properties, particularly its antiviral, anticancer, and anti-inflammatory activities [81]. One of the most well-known bioactive compounds isolated from *P. americana* is pokeweed antiviral protein (PAP). This ribosome-inactivating protein has demonstrated potent antiviral effects by inhibiting viral replication through the suppression of protein synthesis. For instance, PAP has been shown to significantly inhibit Japanese encephalitis virus infection in both in vitro and in vivo models, reducing viral load and enhancing immune response [82]. In addition to its antiviral properties, *P. americana* also exhibits potential anticancer activities. An ethanolic extract of the plant has been reported to show anti-colon cancer activity, with evidence suggesting that it targets cancer cells selectively without causing significant harm to normal cells [83]. Furthermore, another study has demonstrated that Phytolacca-derived carbohydrate products, such as PAP, can inhibit glucose transporters (GLUT1 and GLUT5) in human cells. This has implications for both cancer treatment, as cancer cells are highly dependent on glucose, and for metabolic disorders, where GLUT inhibition can modulate glucose uptake [84]. Recent research has also explored the development of novel fusion antiviral proteins that combine PAP with ricin A chain (RTA), demonstrating enhanced antiviral properties. This fusion protein exhibited a strong ability to inhibit protein synthesis in hepatitis B virus-infected cells, highlighting its therapeutic potential for viral infections [85].

### 3.9. Portulaca oleracea L.

*P. oleracea*, commonly known as purslane or common purslane, is an herb which is found across all of Kentucky and was introduced in North America from Asia [16]. Eastern Kentucky locals have used this plant for general health benefits. It is consumed raw or cooked and then eaten (Table 1).

This is a plant rich in bioactive compounds and has been extensively studied for its wide range of pharmacological properties, including anti-inflammatory, antioxidant, anti-asthmatic, immunomodulatory, anti-tumor, and anti-microbial activities [86]. The phytochemical profile of purslane includes alkaloids, flavonoids, polysaccharides, saponins, and fatty acids, which are responsible for its medicinal benefits [87]. Its ability to suppress oxidative stress and inflammation has been shown to have therapeutic potential in various inflammatory conditions. For example, Rahimi et al. [88] emphasize the plant’s role in modulating pro-inflammatory cytokines, highlighting its capacity to inhibit enzymes such as cyclooxygenase-2 (COX-2), which are involved in the inflammatory response. Purslane’s antioxidants, including vitamins C and E, also play a crucial role in neutralizing free radicals, further reducing oxidative damage and inflammation. Rahimi et al. [88] suggest that the plant’s alkaloids and flavonoids are responsible for these effects, with studies showing that purslane can modulate the expression of genes related to tumor cell survival and metastasis. Furthermore, it has been demonstrated that extracts from *P. oleracea* inhibit hypoxia-inducible factor-1 (HIF-1) and vascular endothelial growth factor (VEGF), which are crucial for tumor growth and angiogenesis [87]. *P. oleracea* is an effective therapeutic agent in gastrointestinal (GI) disorders, particularly colitis. Zhang et al. [89] found that a purslane extract relieves intestinal inflammation by regulating endoplasmic reticulum stress and autophagy pathways, reducing inflammation, and protecting the intestinal mucosa. Similarly, Zhu et al. [90] have demonstrated that exosome-like nanoparticles derived from purslane mitigate dextran sulfate sodium (DSS)-induced colitis by expanding double-positive CD4+CD8+ T cells, suggesting its immunomodulatory role in treating inflammatory bowel diseases. The plant has also been shown to improve symptoms of metabolic syndrome, such as obesity, diabetes, hypertension, and dyslipidemia [91]. The plant’s active compounds help regulate blood glucose levels, reduce lipid profiles, and improve insulin sensitivity. The high fiber content of *P. oleracea*, coupled with its omega-3 fatty acids and antioxidants, makes it effective in combating metabolic disorders by modulating pathways related to glucose metabolism and lipid homeostasis [91].

Purslane is a well-known remedy for respiratory conditions, particularly asthma [92,93,94,95,96]. Khazdair et al. [97] reviewed its anti-asthmatic effects, noting that the plant’s bronchodilator and anti-inflammatory properties can reduce airway hyper-responsiveness and inflammation in asthma patients. Purslane’s alkaloids, such as dopamine, have been shown to act on β2-adrenergic receptors, leading to smooth muscle relaxation in the bronchial tubes, improving breathing and reducing asthma symptoms [98].

*P. oleracea* also exhibits significant antiviral and antimicrobial activities. Zhou et al. [99] have demonstrated that polysaccharides from purslane inhibit porcine rotavirus infection in vitro, suggesting its potential use in antiviral therapies. Additionally, Rahimi et al. [88] highlighted the plant’s antimicrobial properties, with specific reference to its inhibitory effects on bacteria and fungi. These antimicrobial properties are crucial, particularly in the context of rising antibiotic resistance. Purslane’s ethanol and aqueous extracts have shown activity against various pathogenic microorganisms, making it a promising candidate for natural antimicrobial agents.

Lastly, *P. oleracea* has also been found to be effective in the treatment of skin conditions, particularly acne vulgaris. Its antibacterial properties against *Cutibacterium acnes*, the bacteria responsible for acne, as well as its anti-inflammatory effects, make it suitable for reducing acne outbreaks and skin irritation [87]. Moreover, the plant’s rich vitamin and mineral content supports skin repair and regeneration. This is why a number of studies report the benefit of purslane in atopic dermatitis [100,101,102], eczema [103,104], skin and skin barrier damage [105,106], and wounds [107,108,109].

### 3.10. Sassafras albidum (Nutt.) Nees

This tree, commonly called sassafras, is a member of the Lauraceae family. It is found in open woodlands and along roadsides all over Kentucky [16]. It has been used for medicinal purposes since before the 1850s, particularly in the treatment of atopic dermatitis and syphilis. Historical records suggest that Indigenous communities used sassafras to reduce swelling, pruritus and rash of atopic dermatitis [25]. Syphilis, caused by *Treponema pallidum*, manifests in its primary stage as painless chancres. The leaves of the sassafras tree were boiled into tea and administered to patients to induce diaphoretic and laxative effects, helping to prevent the progression of the infection [25]. This was crucial, as unchecked syphilis could advance to neurosyphilis and other severe symptoms of tertiary syphilis.

Sassafras oil, extracted from the roots and bark of the plant, has historically been used to treat pain, particularly neuralgia. In the late 19th century, oil of sassafras was recommended for treating neuralgia due to its ability to relieve nerve pain [26]. Its analgesic effects are thought to be linked to the presence of safrole, a compound known to act as a mild anesthetic. The oil provided temporary relief from localized pain, particularly in dental applications, making it a popular remedy in early traditional medicine.

Sassafras roots are traditionally used as a tonic in a number of disorders [16]. The root bark is also prepared into a tea and employed as diaphoretic, stimulant, diuretic, and carminative [14]. Sassafras oil is used as a flavoring agent in beverages, baking, and toothpaste. It is used in Appalachia to treat bronchitis, with the bark reported to be used for its insect repellent and smoking cessation property while the leaves are used as a dye to make a soft yellow tan [14,15]. Our survey conducted with Eastern Kentucky residents showed that the root is traditionally made into a tea and used for respiratory symptoms (Table 1).

From the scientific literature, we learn that sassafras also shows potential as an antiparasitic agent, particularly against Leishmania [110], which causes leishmaniasis. Monzote et al. [111] explored the antiparasitic activity of various tropical rainforest plant extracts, including those from *S. albidum*. The study found that sassafras exhibited promising antileishmanial activity, suggesting its potential as a natural treatment for parasitic infections. This underscores the plant’s ability to target parasites in the bloodstream, making it a candidate for further research.

Safrole from sassafras root bark [156] has been found to inhibit CYP450 enzymes, which can increase the bioavailability of other drugs when consumed orally. Specifically, CYP1A2, CYP2A6, CYP2E1, CYP3A4, and CYP2D6 are inhibited, affecting pathways such as 7-ethoxyresorufin O-deethylation, coumarin hydroxylation, and chlorzoxazone hydroxylation [157]. Increasing bioavailability is important because it enhances the effectiveness of a single drug. When utilized appropriately, it can reduce the need for multidrug therapies and minimize polypharmacy. Having said that, safrole, ingredient of this herb, is a known carcinogen [110]. This means that there is much caution and concern in terms of its safe use and encouraging the consumption of this plant, keeping in mind the presence of this chemical in it.

### 3.11. Ampelopsis glandulosa (Wall.) Momiy.

Commonly called porcelain, this creeper is native to Asia but found distributed across all of Kentucky [16]. Our survey of local use regarding this plant found that this is traditionally used in the Appalachian culture for its anti-inflammatory effects in arthritis (Table 1). It is also used for its anti-inflammatory, diuretic and hepatoprotective benefits in the Asian folk medicine practices [27,28].

Similar effects have been reported on the pharmacology of this plant in scientific literature. An ethanolic extract of the plant rhizome was tested on mast cells and anaphylaxis. The extract reduced anaphylaxis, extravasation and swelling as measured via hypothermia, histamine and β-hexosaminidase levels, and synthesis of IgE. The extract also suppressed intracellular Ca2+ and downregulated TNF-α and IL-4 via the prevention of the stimulation of nuclear factor-κB (NF-κB) [112]. The extract also inhibited [28,113] immunoglobulin (Ig) E, IgG2a, CD4+IFN-γ+ and CD4+IL-4+, TNF-α, IFN-γ, IL-4, IL-13, IL-6 [114], and IL-1β. In another study, porcelain extract reduced protein and mRNA levels of chemokines, and cytokines exposed by TNF-α/IFN-γ, both in vitro and in vivo, indicating its benefits in atopic dermatitis [115]. Chang et al. [116] reported the presence of a resveratrol derivative in the plant, vitisinol-A, that has potent anti-inflammatory activity, as measured in the 3-(4,5-dimethylthiazol-2-yl)-2,5-diphenyltetrazolium bromide (MTT) assay and lipopolysaccharide (LPS)-stimulated RAW264.7 cells.

In addition, the extract has also been reported to have effects that aid in wound healing via its antioxidant and anti-inflammatory potential. A porcelain extract increased cell migration rate, reduced inflammation, increased epithelization and angiogenesis, and helped in deposition of collagen [117,118]. A methanolic extract of *A. glandulosa* has also been shown to demonstrate the potent scavenging effect of hydroxyl radicals, thus indicating its antioxidant potential [27]. This antioxidant effect of the plant, along with its anti-inflammatory potential, is responsible for its hepatoprotective behavior, as it is also used in folk medicine. When tested in hepatic stellate cells in vitro, an ethanolic extract of the plant induced apoptosis and suppressed the expression of liver fibrosis-related genes. When tested in vivo in rat, the extract pretreatment reversed serum hepatic biomarkers, halted abnormal histopathological observations and corrected glutathione and hydroxyproline levels [119]. Similar results have been reported by Yabe and Matsui [120], who used an aqueous extract of porcelain berry in mice. The extract inhibited hepatic injury, induced by carbon tetrachloride and D-galactosamine [121], as determined by centrilobular necrosis, cytoplasmic vacuolation, cellular swelling, inflammation, fibrosis, reactive oxygen species [122,123,124] and collagen accumulation [124].

Finally, the plant is also exhibited to have other benefits. Resveratrol derivatives, hopeaphenol and vitisin-A, isolated from porcelain, inhibit angiotensin 1 converting enzyme (ACE), indicating an antihypertensive effect of the plant [125]. In terms of bone loss prevention, an extract of porcelain showed significant anti-osteoclastogenic activity both in vitro and in vivo [126]. The extract inhibited RANKL-induced osteoclast differentiation, actin ring formation, and bone resorption and inhibited lipopolysaccharide (LPS)-induced bone erosion [126]. The extract has been also shown to have antimutagenic [127] and antiviral [128] effects.

### 3.12. Ulmus rubra Muhl.

*U. rubra* (slippery elm) is found frequently in Kentucky, on dry slopes and in moist, well-drained bottomlands [15]. It has been traditionally used for various medicinal purposes, particularly for its soothing effects on the GI tract. The bark contains mucilage, which becomes gel-like when mixed with water, creating a protective coating that alleviates irritation in the throat, esophagus, and stomach [29]. This property makes slippery elm effective in treating conditions like gastroesophageal reflux disease (GERD) and irritable bowel syndrome (IBS). Mucilage from the soaked bark is also traditionally used as a protective, anti-inflammatory, skin softener, laxative, and wound healing agent [14,15]. Our survey showed that local Eastern Kentuckians have traditionally used tea of the bark of this plant for laxative purposes (Table 1).

In a study by Ried et al. [129], slippery elm was included in an herbal formula that significantly improved both upper and lower GI symptoms in adults with digestive disorders, highlighting its potential role in promoting gut health [129,130].

Beyond its GI applications, *U. rubra* also exhibits anti-inflammatory and antioxidant properties. Compounds such as catechins and flavonoid glycosides found in the *Ulmus* genus have shown promise in managing inflammatory conditions and even osteoporosis [131]. *Ulmus* extracts regulate the activity of osteoclasts through the RANKL, which improves bone density and strength [131]. Additionally, recent research suggests slippery elm’s potential role in enhancing intestinal barrier function. Aleman et al. [132] found that yogurt made with starter culture bacteria and supplemented with ingredients such as slippery elm bark were able to treat leaky gut. Specifically, *U. rubra* helps strengthen tight junctions, protecting the infrastructure of the intestines.

### 3.13. Verbascum thapsus Linnaeus

*V. thapsus* (commonly known as mullein) has been traditionally used for various medicinal purposes, with modern research supporting its healing properties. It is a naturalized plant from Europe but found abundantly in Eastern Kentucky [17]. Tea made from mullein leaves and flowers is traditionally known to have astringent, cough and pain suppressing (use for cold and flu), and antimicrobial properties. Locals from New Mexico smoked the dried leaves to help with symptoms of asthma [14]. The findings of the survey showed that the local population of Pike County, Kentucky have used this plant traditionally for its cough suppressant properties. Locals use any parts of the plant and make it into tea (Table 1).

Looking at the published data, a randomized controlled trial by Taleb and Saeedi [133] demonstrated that *V. thapsus* significantly improved episiotomy wound healing in nulliparous women, accelerating tissue repair and reducing pain. This supports the idea of its role as a natural remedy for promoting skin healing. Additionally, its antioxidant properties have also been highlighted in [134], which found that bioactive compounds from *V. thapsus* roots exhibited potent antioxidant activity, suggesting its potential in protecting against oxidative stress-related diseases. Other groups have also reported antioxidant [135,136,137,138], antimicrobial [139], antiviral [140,141], and anticancer [142,143] properties.

*V. thapsus* also shows promise in addressing parasitic and inflammatory conditions. Ali et al. [144] discovered its anthelmintic and spasmolytic activities, indicating the plant’s effectiveness in treating parasitic worm infections. Meanwhile, Calabrese et al. [145] found that *V. thapsus* leaf extract demonstrated anti-inflammatory and anti-osteoarthritic properties, showing its potential as a therapeutic agent for inflammation and joint conditions. Other studies have also shown the anti-inflammatory potential of mullein [146]. Furthermore, Fakhrieh-Kashan et al. [147] noted that a combination of *V. thapsus* and ginger extract induced apoptosis in Trichomonas vaginalis, further suggesting its use in treating parasitic infections. The antiparasitic effect of mullein is widely reported [148,149].

### 3.14. Xanthorhiza simplicissima Marshall

In the early nineteenth century, the shrub *X. simplicissima*, or yellowroot as it is commonly called, was used as a dye to color ceremonial feathers by the Cherokee people [15,16,30]. Native indigenous people used *X. simplicissima* root infusion as a tonic for cramps, dyspepsia, hemorrhoids, sore eyes, sore throat, stomach ulcers, colds, and jaundice [14,20,30]. Conversations with Eastern Kentucky locals, as part of this survey, revealed that a bitter liquid made from the roots of the plant is used in sore throat (Table 1).

*X. simplicissima* was historically valued in medicine due to its bitter taste, largely attributed to the presence of alkaloids such as berberine [158]. Berberine is the major active component of *X. simplicissima*, while puntarenine is another alkaloid which was extracted from the plant [150]. Berberine exhibits activity against AIDS-related pathogens such as *Candida albicans*. *Cryptococcus neoformans*, and *Mycobacterium intracellularae*, while puntarenine showed activity against *Trichophyton mentagrophytes* and *Saccharomyces cerevisiae* [150]. Studies have shown that *X. simplicissima* also has potential cytotoxic effects, pointing towards its anticancer benefits [151].

## 4. Limitations of This Study

One major limitation of this study is the small number of survey participants. We were only able to identify and interview four individuals who had some knowledge of local medicinal plants in Pike County, Eastern Kentucky. All four participants were University of Pikeville employees, although they are long-term locals of the area and have family roots going back several generations. This represents a form of sampling bias, as the participants do not fully reflect the broader community and their experiences may differ from people living out in the community and are not part of the University. Efforts to locate practicing herbalists, traditional healers, or elders with extensive plant knowledge were unsuccessful. This likely reflects the gradual loss of traditional herbal knowledge in the region, as fewer people now practice or pass down this information. One of the goals of this study is to raise awareness around this topic in this area of the country so that measures can be taken to revitalize the acquisition and preservation of this knowledge.

Another limitation of the study is its restricted geographic focus. The research was conducted only in Eastern Kentucky, within the Central Appalachian region. While this area has a rich history of plant-based knowledge, limiting the study to one part of Appalachia means that important regional differences in medicinal plants could not be captured. Further studies are planned in the future to expand on this initiative, and to include other parts of the Appalachian region to document a wider range of plants and medicinal plant use practices. Such work would help build a more complete understanding of traditional medicinal knowledge and could present additional, lesser-known plant uses that are still preserved in other local communities.

## 5. Conclusions

This comprehensive review highlights the remarkable diversity and therapeutic potential of medicinal plants native to Eastern Kentucky. The findings present a list of 14 medicinal plants used in Eastern Kentucky, along with details on their contemporary applications. For these plants surveyed and listed, this study reveals the available scientific literature that further substantiates their use. It is clear that these plants demonstrate significant pharmacological activities that align with their traditional uses. In particular, a number of plants were found to have been reported for their anti-inflammatory (*Cichorium intybus*, *Eupatorium perfoliatum*, *Monotropa uniflora*, *Paulownia tomentosa*, *Phytolacca americana*, *Portulaca oleracea*, *Ampelopsis glandulosa*, *Ulmus rubra*, *Verbascum thapsus*), antimicrobial (*Asimina triloba*, *Callicarpa americana*, *Chimaphila umbellate*, *Cichorium intybus*, *Eupatorium perfoliatum*, *Paulownia tomentosa*, *Phytolacca americana*, *Portulaca oleracea*, *Sassafras albidum*, *Ampelopsis glandulosa*, *Verbascum thapsus*, *Xanthorhiza simplicissima*), and antioxidant (*Asimina triloba*, *Chimaphila umbellate*, *Cichorium intybus*, *Paulownia tomentosa*, *Portulaca oleracea*, *Ampelopsis glandulosa*, *Ulmus rubra*, *Verbascum thapsus*) properties. Other activities like anticancer, bone protective, hepatoprotective, antidiabetic, antiallergic, analgesic, antigout, immunomodulatory, cardioprotective, antihypertensive, neuroprotective, anti-asthmatic, wound healing, and skin protective were also noted. Moreover, this study reveals the need for further research to fully understand the mechanisms of action and potential applications of these plants. While traditional knowledge has provided valuable insights into their therapeutic applications, modern scientific investigation is essential to validate these uses and ensure safety and efficacy. The presence of multiple bioactive compounds in many of these plants suggests potential synergistic effects that warrant further investigation. Additionally, the sustainable harvesting and conservation of these valuable medicinal plants should be prioritized to ensure their availability for future generations while preserving the rich botanical heritage of Eastern Kentucky.

## Figures and Tables

**Table 1:** List of traditionally used plants surveyed for their medicinal use in Pike County, Eastern KY (data gathered through a survey of some Appalachian locals revealed 14 plants, all listed in the table below).

Scientific Name	Family	Plant Type	Plant Local Name	Medicinal Use of Plant	Plant Part Used for Consumption	Method of Preparing Plant for Medicinal Use	References for Plant Details
*Asimina triloba*	Annonaceae	Tree	Pawpaw	Used as a food	Fruit	Eaten as is (tropical fruit)	[15,16,17,18]
*Callicarpa americana*	Lamiaceae	Shrub	Beautyberry	Insect repellant	Berry		[16,19,20]
*Chimaphila umbellata*	Ericaceae	Shrub	Pipsissewa	Antirheumatic	Leaf		[21,22]
*Cichorium intybus*	Asteraceae	Herb	Chicory (blue)	Coffee alternative	Root	Made into tea	[16,19,20]
Eupatorium perfoliatum *	Asteraceae	Shrub	Boneset	Laxative, cough	Leaf	Made into tea	[14,16,19,20,23,24]
*Monotropa uniflora*	Ericaceae	Herb	Ghost pipe	Pain relief	Flower		[16,17]
*Paulownia tomentosa*	Paulowniaceae	Tree	Princess tree	As a food/salad	Fruit	Fruit sliced and eaten	[15,16,17]
Phytolacca americana *	Phytolaccaceae	Herb	Pokeweed	Anti-arthritic	Berry	Poisonous (seed; sap allergy causing) but made into edible via boiling. Berry made into jam	[14,16,17,19,20]
Portulaca oleracea *	Portulacaceae	Herb	Purslane	Overall health	Leaf	Edible raw or cooked	[16]
Sassafras albidum *	Lauraceae	Small tree	Sassafras	Respiratory (bronchitis)	Root	Made into tea	[14,15,16,25,26]
Ampelopsis glandulosa *	Vitaceae	Creeper	Porcelain berry	Anti-arthritic	Berry		[16,27,28]
Ulmus rubra *	Ulmaceae	Tree	Slippery elm	Laxative	Bark	Made into tea	[14,15,29]
*Verbascum thapsus*	Scrophulariaceae	Herb	Mullein	Expectorant	Whole plant	Made into tea	[14,17]
Xanthorhiza simplicissima *	Ranunculaceae	Shrub	Yellowroot	Sore throat	Root	Root made into a bitter liquid	[14,15,16,20,30]

* These plants have synonyms (alternative scientific name), which are given in Table 2.

**Table 2:** Synonyms (alternative scientific name) for some of the medicinal plants that are part of the findings of the survey.

Plant Names	Alternate Scientific Names
*Eupatorium perfoliatum*	*E. chapmanii*, *E. connatum*, *E. salviifolium*, *E. truncatum*, *E. cuneatum*, *Uncasia cuneata*, *U. truncata*, *U. perfoliate*, *Cunigunda perfoliata*
*Phytolacca americana*	*P. decandra*, *P. rigida*
*Portulaca oleracea*	*P. hortensis*, *P. officinarum*
*Sassafras albidum*	*S. officinalis*, *S triloba*, *S. variifolium*, *Laurus sassafras*
*Ampelopsis glandulosa*	*A. brevipedunculata*, *A. citrulloides*, *A. heterophylla*, *A. regeliana*, *A. sinica*, *Cissus brevipedunculata*, *Vitis brevipedunculata*, *V. elegans*, *V. glandulosa*, *V. heterophylla*, *V. sinica*
*Ulmus rubra*	*U. americana*, *U. crispa*, *U. dimidiate*, *U. elliptica*, *U. fulva*, *U. heyderi*, *U. pinguis*, *U. pubescens*
*Xanthorhiza simplicissima*	*X. apiifolia*

**Table 3:** Reported pharmacological benefits of Eastern Kentucky medicinal plants.

Plant Name	Reported Activities in Literature	References
*Asimina triloba*	Antioxidant; anticancer; antimicrobial	[31,32,33,34,35]
*Callicarpa americana*	Antimicrobial; insect repellant	[36,37,38,39,40]
*Chimaphila umbellata*	Antifungal; anticancer; antioxidant; bone protective; antimicrobial	[21,22,41,42,43]
*Cichorium intybus*	Hepatoprotective; antiallergic, antitumor, antioxidant, anti-inflammatory; antidiabetic; antimicrobial; analgesic; antiprotozoal; anti-gout	[44,45,46,47,48,49]
*Eupatorium perfoliatum*	Immunostimulant; antiviral; cardioprotective; anti-inflammatory; cytotoxic & antibacterial	[50,51,52,53,54,55,56,57,58]
*Monotropa uniflora*	Neurotransmission regulator; pain relief	[59,60]
*Paulownia tomentosa*	Antioxidant, antibacterial, cytotoxic; anti-inflammatory; anticancer, antimicrobial; antiv iral, neuroprotective	[61,62,63,64,65,66,67,68,69,70,71,72,73,74,75,76,77,78,79,80]
*Phytolacca americana*	Antiviral, anticancer, anti-inflammatory; antidiabetic	[81,82,83,84,85]
*Portulaca oleracea*	Antioxidant, anti-inflammatory, anti-asthmatic, immunomodulatory, anticancer, antimicrobial; GI health; anti-metabolic syndrome; antiviral; wound healing	[86,87,88,89,90,91,92,93,94,95,96,97,98,99,100,101,102,103,104,105,106,107,108,109]
*Sassafras albidum*	Antiparasitic	[110,111]
*Ampelopsis glandulosa*	Anti-inflammatory; wound healing; antioxidant; hepatoprotective, antihypertensive; bone protective; anticancer; antiviral	[28,112,113,114,115,116,117,118,119,120,121,122,123,124,125,126,127,128]
*Ulmus rubra*	GI health; antioxidant, anti-inflammatory; bone protective	[129,130,131,132]
*Verbascum thapsus*	Wound healing; antioxidant; antimicrobial; antiviral; anticancer; anti-inflammatory, anthelmintic, antispasmodic; bone protective; antiprotozoal	[133,134,135,136,137,138,139,140,141,142,143,144,145,146,147,148,149]
*Xanthorhiza simplicissima*	Antimicrobial, anticancer	[150,151]

## Data Availability

All data are presented in this manuscript.
